# Variation in Phytochemical, Antioxidant and Volatile Composition of Pomelo Fruit (*Citrus grandis* (L.) Osbeck) during Seasonal Growth and Development

**DOI:** 10.3390/plants10091941

**Published:** 2021-09-17

**Authors:** Arun Kumar Gupta, Subhamoy Dhua, Partha Pratim Sahu, Giulia Abate, Poonam Mishra, Andrea Mastinu

**Affiliations:** 1 Department of Food Engineering and Technology, Tezpur University, Tezpur 784028, Assam, India; arunk@tezu.ernet.in (A.K.G.); subhamoydhua94@gmail.com (S.D.); 2 Department of Electronics and Communication Engineering, Tezpur University, Tezpur 784028, Assam, India; pps@tezu.ernet.in; 3 Department of Molecular and Translational Medicine, Division of Pharmacology, University of Brescia, 25123 Brescia, Italy; andrea.mastinu@unibs.it

**Keywords:** pomelo, maturity, volatile compounds, tannin, naringin, *Citrus grandis*

## Abstract

Citrus fruits exhibit a high level of different phytoconstituents, of which the changes in the different parts of the fruit during ripening have not been thoroughly studied yet. Thus, in this study, we have investigated how different parts of pomelo fruit (*Citrus grandis* L.) are modified throughout the development of two consecutive growing seasons. In detail, the main phytochemical compounds, such as total phenolic content, total flavonoid content, antioxidant capacity, DPPH free radical scavenging activity, Ferric reducing antioxidant power (FRAP), and naringin and tannin content, were analyzed. A systematic metabolism of these compounds was found during the development of the fruit, but some pomelo tissues showed a fluctuating trend, suggesting a dependence on the different growing season. Focusing on the tissue distribution of these compounds, the fruit membrane contained the highest level of total phenolic and flavonoid content; fruit flavedo displayed the highest antioxidant capacities and FRAP activities, whereas maximum accumulation of naringin was noticed in fruit albedo. Instead, the highest DPPH free radical scavenging activity and tannin contents were found in the pomelo juice. Regarding the distribution of compounds, a possible bias pattern for the accumulation of those compounds has been noticed throughout the fruit development. From the GC-MS analysis, a total of 111 compounds were identified, where 91 compounds were common in both seasons. Overall, these results could be useful for the food processing industry as guidelines for excellent quality foods and for introducing health-beneficial products and components into our daily diets.

## 1. Introduction

Fruits and vegetables are rich in numerous bioactive compounds which have shown many applications in human well-being [1,2,3,4]. Additionally, the nutritional value of fruit and vegetables has gained interest for consumers, as they play a key role in disease prevention [5,6]. They are consumed mainly for their nutritional properties and quality, which are essential parameters to attract consumers and define their market potential.

Pomelo (*Citrus grandis* (L.) Osbeck), also known as pummelo, shaddock, or Chinese grapefruit, is commonly classified as common (or white) or pigmented (or pink) [7,8]. According to data released from FAO (Food and Agriculture Organization of United Nations), the worldwide production of pomelo is about 9.3 million metric tons. China and the United States are the leading pomelo producers around the world, with approximately 5.0 and 0.5 million metric tons, respectively (FAOSTAT, 2019). In Southeast Asia and other parts of the world, pomelo has been growing in popularity and is now one of the five most widely cultivated and consumed citrus fruits, along with orange, mandarin, lemon, and grapefruit [9]. It tastes sweet and is slightly acidic with a hint of bitterness. Although some comparisons have been made on the volatile fractions of Nakon pummelo [10] and Chandler pummelo [11], differences in phytochemical composition of the different parts of pomelo fruits are still unexplored [12,13].

Citrus fruit composition varies significantly due to fluctuating effects, such as rootstock, fruit size, variety, maturity, storage, horticultural conditions, and climate, suggesting that nutrient and constituent analysis only provide within- and between-variety estimates, and general conclusions are difficult to draw [14]. Among foods, citrus fruits are major sources of β-cryptoxanthin for the human diet. In addition, citrus fruits are also a source of carotenoids (for example, β-cryptoxanthin) and flavonoids [15].

Phytochemicals including polyphenols found in plant materials have gained interest due to their scavenging activity and antioxidant properties responsible for anti-inflammatory, anticarcinogenic, antiatherogenic, antithrombotic, immune-enhancement, and vasodilatory effects, which are just a few of their many health beneficial effects [16].

Flavonoids have a polyphenol structure and are responsible for the flavor in many fruits and vegetables [17] and may act as defensive agents for the biotic and abiotic stress of the plants [18,19,20,21,22,23,24,25]. Flavonoids are considered secondary metabolites of plants and have many possible health benefits when consumed [26]. One of the most common classes of flavonoids found in citrus fruits is naringin, which gives grapefruit its bitter taste [17].

Ripeness or maturity index is considered a key factor that can strongly imbalance physiological factors (color, shape, size, and texture) and nutritional value of fruits [27]. Previous authors have documented how antioxidant properties vary at different maturity stages in fruits, such as olive, orange, tomato, and pear jujube [6,28,29,30,31].

Generally, there is a lack of knowledge for the relationship between polyphenols content, soil properties, and agricultural practices. For economic reasons, intensive agriculture favors the rapid development of fruits in plants’ growth in highly nutritious soils, compared to the production of fruits with a higher secondary metabolite content. Studying changes in the nutritional value of fruit during the ripening process can help estimate the optimal date for fruit harvesting to achieve the best quality for direct consumption and further use. A better understanding of the biochemical changes occurring in medlar, the chemistry of phytochemical transformations in fruits and their functions in plant physiology should stimulate interest in maximizing the beneficial sensory, and nutritional effects of polyphenols in the diet. Thus, if this variation is mapped, it will allow indirect estimation of the antioxidant content of the citrus without time-consuming laboratory measurements, and immediate identification of ready-to-harvest trees [32].

To the best of our knowledge, there are not enough data about changes in phytochemical and total antioxidant activity during the development and maturation of different parts of pomelo fruit.

Therefore, the aim of this study is to understand the phytochemical variation that occurs in the two consecutive seasons in the different parts of the pomelo along with mapping the overall flavor quality-related chemicals in the juice. This will provide comprehensive information to the juice processing and pharmaceutical industry to select the optimal harvest time and use the best parts of pomelo fruit for the extraction of bioactive compounds.

Overall, the results here presented could be useful for the food processing industry as guidelines for excellent quality foods and for introducing health-beneficial products and components into our daily diets.

## 2. Materials and Methods

### 2.1. Materials

**Figure 1:**
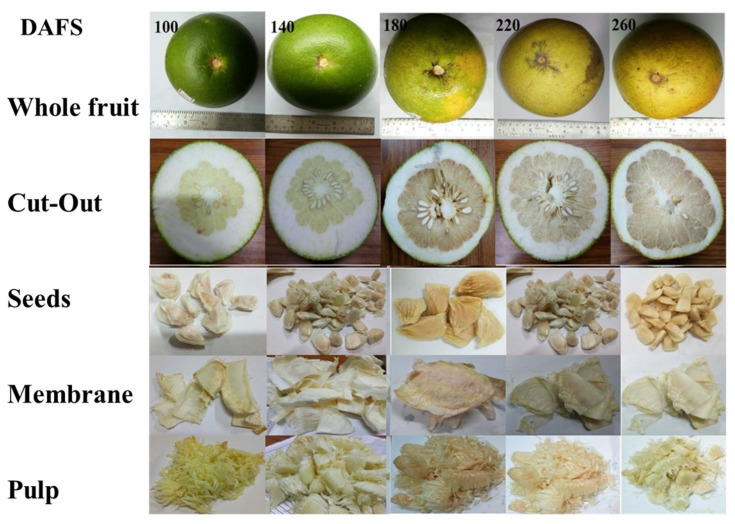
Pomelo fruit (whole; cut out section; and various parts of pomelo fruit at various maturity stages.

Pomelo fruit (*Citrus grandis* L. Osbeck var. Robab tenga) at different maturity stages was collected from the horticulture department of Tezpur University, Assam, India (Figure 1). All the reagents used for analysis were of analytical grade and procured from Merck Millipore, India.

### 2.2. Sample Collection

The pomelo fruit was studied in two consecutive seasons i.e., (i) from April–October 2018 and (ii) from April–October 2019. The experiment was composed of five collection periods. The pomelo fruits were collected in the morning from tagged six healthy trees from 100 days after fruit set (DAFS) to 260 DAFS with an interval of 40 days and divided into three equal batches, representing the replicates. In each stage, 25–30 fruits were collected for the experiment.

### 2.3. Juice Extraction and Extract Preparation of Seeds, Membrane, Albedo, Flavedo, and Pulp

The fruit was peeled where flavedo was separated from albedo using a sharp knife, whereas different parts, such as seed, membrane, and pulp, were carefully separated using a scalpel and tweezers (Figure 1). The juice was extracted using a laboratory-scale juicer (Philips HR1832/00) and filtered by a three-layered cheesecloth. The juice samples were centrifuged at 7000 rpm for 10 min to produce a clear solution and stored in a screw-capped bottle at 5 °C for further analysis. On the other hand, the paste of different parts of fruits was prepared using a laboratory blender and extraction was performed using methanol and water in 80:20 (*v*/*v*) [3]. The prepared mixture was placed in the incubator shaker at 37 °C for 24 h. Extracts were then centrifuged at 7000 rpm for 15 min and the supernatant was collected in the vials and stored at 5 °C [3] for further analysis of the phytochemical property and naringin estimation.

### 2.4. Spectrophotometric Determination of Phytochemical Properties

Total phenolic content present in the different parts of pomelo fruit was estimated according to Mishra and Kalita (2017) [33]. The gallic acid was used as a standard and results are presented in terms of gallic acid equivalent (GAE) (µg GAE g^−^^1^ or mL^−^^1^). Total flavonoids were measured using a colorimetric assay adapted from Zhishen, Mengcheng, and Jianming (1999) [34]. Quercetin was used as standard and the obtained results are expressed in terms of quercetin equivalent (QE) (µg QE g^−^^1^ or mL^−^^1^). Condensed tannin was calculated by the vanillin essay, based on the procedure reported by Broadhurst and Jones (1978) [35] and results are expressed as mg catechin equivalent (CE) per g of peel or juice. Hydrolysable tannin was calculated as per Bossu et al. (2006) [36]. The standard curve (0–5 mg mL^−^^1^ of tannic acid) was used for the calculation of hydrolysable tannin in peel or juice.

### 2.5. Determination of Naringin Content

The extract of different parts of fruit and juice were analyzed for naringin content using the HPLC (Waters Corporation, Milford, MA, USA) system equipped with a C-18 column-reversed phase (5 μm) (7.2 × 300 mm^3^), with HPLC pump-515, loop injector system, enabled with EMPOWER-2 software. In the present investigation, we extracted the total phenolic contents including naringin using 80% methanol for analysis for both FCR and HPLC. The extract was diluted with acetonitrile (40%) in the ratio of 1:1 followed by filtration using a 0.45 μM PTFE membrane, and the filtered solution (20 μL) was injected. The solution used for mobile phase A was potassium dihydrogen phosphate (10 mM), while acetonitrile (70%) was kept as mobile phase B and detection was carried out in a gradient mode at 280 nm [37] using a UV Visible detector.

### 2.6. Ferric Reducing Antioxidant Power (FRAP), DPPH Free Radical Scavenging, and Antioxidant Capacity

FRAP was carried out according to Benzie and Strain (1996) [38], where extract was mixed with FRAP reagent (2.7 mL) and topped of to 3 mL with distilled water. The mixture was allowed to react in the dark for 30 min at 37 °C. The absorbance of the solution was recorded at 593 nm, and the results are expressed in terms of mM FeSO_4_ g^−1^ or mL^−1^ of the sample, whereas DPPH free radical scavenging activity of pomelo fruit was determined as per the method of Blois (1958) [39]. In brief, the extracts (0.5 mL) were mixed with 3 mL of DPPH solution in methanol (0.1 mM). The prepared mixture was allowed to react in the dark at 37 °C for 30 min, and absorbance was measured at 517 nm. Ascorbic acid was used as a positive control.

(1) $$\mathrm{DPPH}\;\mathrm{free}\;\mathrm{radical}\;\mathrm{scavenging}\;\mathrm{activity}\;\left(\%\;\mathrm{inhibition}\right)=\frac{A_{c}-A_{s}}{A_{c}}\times 100$$

where A_c_ = Absorbance of control; A_s_ = Absorbance of sample

The antioxidant capacity by the phosphomolybdenum method of pomelo fruit was evaluated according to the method described by Jayaprakasha et al. (2002) [40]. Briefly, extract (100 μL) was mixed with 1 mL of freshly prepared reagent solution (0.6 mol L^−1^ sulfuric acid, 28 mM L^−1^ sodium phosphate, and 4 mmol L^−1^ ammonium molybdate). Extract solution (80% methanol and 20% water) was used as blank. The mixture was vortexed and screw capped. The tubes were incubated for 90 min at 95 °C in a boiling water bath. The tubes were allowed to cool at room temperature and the developed green color of the solution was read at 695 nm against a blank. Total antioxidant capacities of the pomelo fruit extract were expressed in terms of ascorbic acid equivalents (mM g^−1^ or mL^−1^ of sample).

### 2.7. Extraction of Volatile Compounds and GC-MS/FID Analysis

The maturity effect on the volatile composition of pomelo juice was identified according to Cheong et al. (2012) [13], where 10 mL of juice was spiked with standard 5-methyl-2-hexanone (100 µL of 10,000 ppm) and was extracted twice with dichloromethane (8 mL). The mixture was vortexed for 30 min followed by centrifugation at the speed of 7000 rpm for 5 min at 4 °C After the centrifugation, the gel was broken up using a glass rod and the organic phase was collected, which was further dried with anhydrous sodium sulphate. The solution was filtered using a Whatman syringe filter (0.2 µm) and concentrated in the presence of a gentle stream of nitrogen to a final volume of 0.10 mL. All the prepared extracts were kept at −20 °C before determination. Analyses of the volatile compounds of pomelo juices were carried out using gas chromatography (GC) equipped (Clarus 600, PerkinElmer, Waltham, MA, USA) with FID and a 5975 inert MSD with software (Turbomass-Ver). In brief, extract (1 µL) was injected directly into the GC injector under splitless mode. NIST 8.0 MS library was used to identify the volatiles by comparing the mass spectra (National Institute of Standards and Technology, Gaithersburg, MD, USA), and the amount was quantified by calculating the area under the curve of a particular component; however, due to the unavailability of standards, the same could not be authenticated and, therefore, the concentration of the components is not reported here.

### 2.8. Statistical Analysis

The data were analyzed statistically using package IBM SPSS Statistics Version 20.0, Armonk, NY: IBM Corporation, and the means were separated using Duncan’s multiple range test (*p* < 0.05). For each experiment, five replicates have been taken for the calculation and all the data are presented as the mean with the standard deviation. In addition, correlations between DAFS and bioactive compounds were examined by bivariate analysis to determine the rhythm with maturity days [41].

## 3. Results and Discussion

Pomelo fruit commonly grown in Asian countries have a zest of bitterness, and its acidic nature was studied consecutively for two years, in which more emphasis was placed on changes in phytochemical composition during the growth and development of various fruit parts, such as seed, membrane, albedo, flavedo, pulp, and juice. The main phytochemical properties displayed a decreasing trend over the maturity throughout the development of fruit parts. The fruit harvested in 2018 had a higher total phenolic content, total flavonoid content in the membrane, and antioxidant activity and FRAP in flavedo compared to 2019. In addition, abundant naringin was found in albedo, whereas DPPH free radical scavenging activity in the juice was observed. However, peel and juice displayed the highest condensed tannin and hydrolysable tannin, respectively. An almost similar decreasing trend was observed in different parts of fruits harvested in 2019 where abundant FRAP, total phenolic content, and antioxidant capacity were found, especially in flavedo. Seed showed higher total flavonoid content, while albedo registered higher naringin content. Juice had the highest DPPH free radical scavenging activity.

### 3.1. Phytochemical Content

#### 3.1.1. Total Phenolic Content

**Figure 2:**
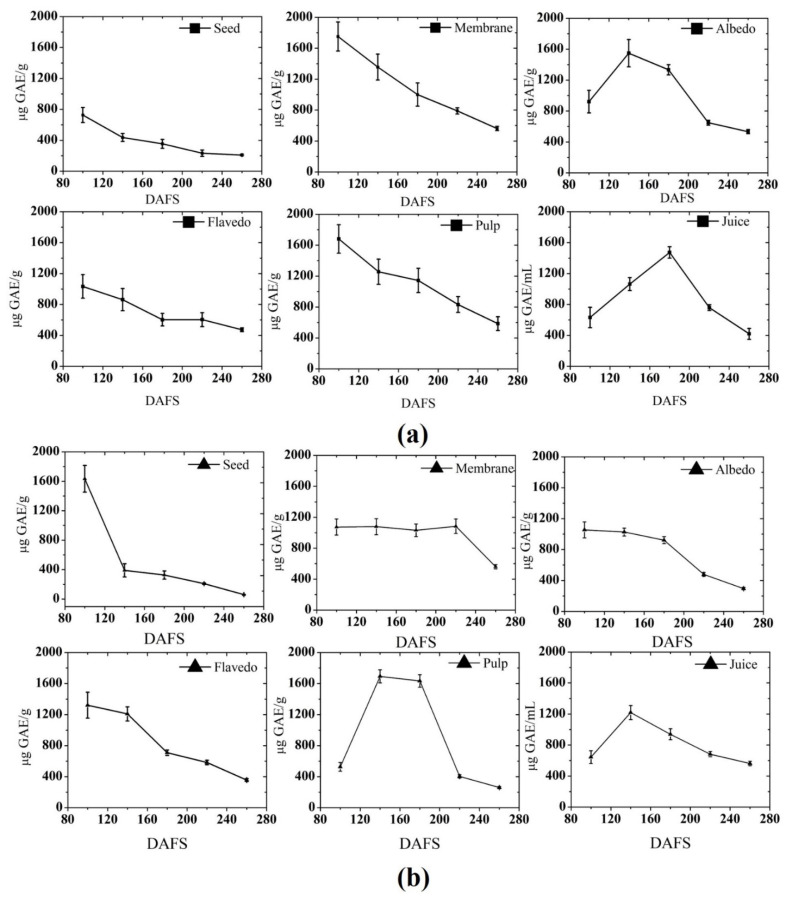
Total phenolic content in (**a**) 2018; and (**b**) 2019. DAFS; days after fruit set.

Results showed a significant difference (*p* < 0.05) in TPC of different parts of pomelo fruit over maturity (Figure 2). The highest phenolic content was observed in the early stages of seed germination and the lowest content was detected in over-ripening stages (over 220 DAFS). TPC content among all parts was found maximum in the membrane of fruits harvested in 2018 (1752 μg GAE g^−1^) than those in 2019 (1074 μg GAE g^−1^). However, changes in the membrane were identical in both years. In the albedo, maximum TPC was detected at 140 and 100 DAFS in 2018 and 2019, respectively. A notable increasing pattern was seen in 2018, leading to a higher content of phenol in the albedo than that of 2019. Flavedo contains maximum essential oil, shows maximum TPC (1033 μg GAE g^−1^) at 100 DAFS in 2018 and 1321.5 μg GAE g^−1^ in 2019 at 100 DAFS followed by a decline of phenol content with maturity. However, a steeping pattern was observed throughout the development stage of flavedo. Noteworthy, in the pulp, an increasing stepping pattern was seen, leading to a maximum TPC of 1680 μg GAE g^−1^ in 2018, while a stagnant period was noticed from 120–200 DAFS in 2019. Moreover, the highest content was registered in 2019 as compared to 2018. TPC of the juice was the highest at 180 DAFS (1472 μg GAE mL^−1^) among the two seasons throughout the development stages.

Phenolics (phenolic acids and flavonoids) are secondary metabolites and are a major source of the antioxidant properties of plants [42]. The decreased level of total phenolic content in pomelo fruits with maturity is in agreement with the previous reports, where the content was found to be decreased as they get matured, such as in carambola, pear jujube, and ciku [6,31,42,43]. The variation in the level of phenol could be correlated with expression of polyphenol biosynthetic enzyme genes, polyphenol oxidase (PPO), and phenylalanine ammonia lyase (PAL). The decrease in content of phenol could be associated with the increased activity of PPO and decreased activity of PAL [44,45]. Huang et al. (2007) [29] reported that a decrease in superoxide dismutase, catalase, and guaiacol peroxidase might be another possible reason for the decrease in total phenolic content in the fruit as they mature or ripen. These aforementioned reasons support the variation in the level of phenol content in the fruit during growth and developmental stages.

#### 3.1.2. Total Flavonoid Content

Flavonoids and phenols are the most abundant group of compounds found in plants, which greatly affect the plant during its growth, and their content varies according to the growth of the plant.

**Figure 3:**
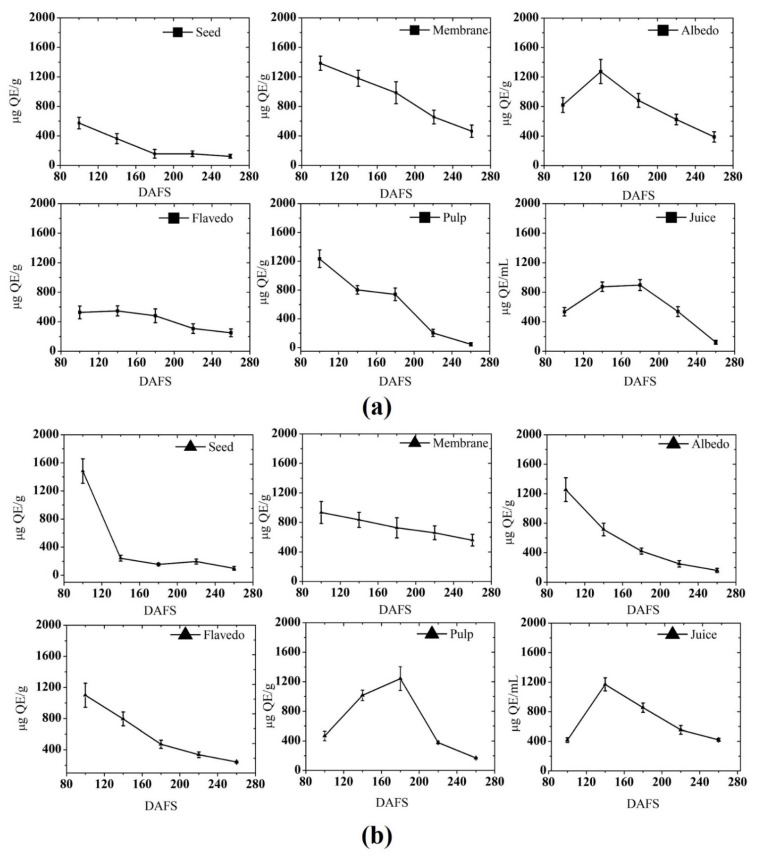
Total flavonoid content in (**a**) 2018; and (**b**) 2019. DAFS; days after fruit set.

Most of the flavonoids (flavanones and flavanone glycosides) found in the citrus plants are rarely present in other plants. The main flavanone glycosides in pomelo, grapefruit, and mandarin species were naringin, hesperidin, and narirutin [45,46,47]. The flavonoid content of pomelo fruit was determined by the aluminum chloride method and the results are presented in Figure 3.

It was found that the membrane has the highest flavonoid content (1384.38 μg QE g^−1^) among all parts of the pomelo during the development phase at 100 DAFS. The content was reported to be higher in pulp (1233.86 μg QE g^−1^) at 100 DAFS, while albedo and flavedo showed a maximum at 140 DAFS in 2018. A regular decline was noticed until the harvest stage in all the fruit’s parts. In 2019, the seed had the highest content (1428.8 μg QE g^−1^), followed by albedo (1258 μg QE g^−1^). The tendencies of total flavonoid content (TFC) changes in both seasons were quite similar. In the pulp, the largest amount of flavonoid was noticed in 2018, which was higher than 2019 throughout the fruit development. The change in content was though not identical in both seasons. In 2019, the accumulation in pulp was increasing until 200 DAFS, where the highest amount of content was observed at 1242.8 μg QE g^−1^. Compared with the levels at the harvest stage, 69–72% of content was detected. In both seasons, the highest content in the juice was detected in 2019, with a level of 1169 μg QE g^−1^, which was 23.69% higher than the previous season. In the beginning stage of fruit development, the flavedo accumulated the highest amount of content in both 2018 and 2019. However, the highest content among both seasons was noticed in 2018, and subsequently, it underwent a regular decline until 260 DAFS. In general, flavonoid content decreased until reaching the overripe stage (260 DAFS), which is in agreement with previous studies where a similar variation in flavonoid content was observed. Samaniego et al. (2020) [48] reported lower levels of TFC at the complete maturity stage in the blackberry. Ersus and Cam (2007) [49] also reported a decreased concentration of flavonoids in citrus with maturity. The reason for significant variations in content could be the balance between biosynthesis and metabolism, which correlates with the progression of maturity.

Flavonoids are, in turn, present at lower concentrations, since they are part of the TPC, and their content also varies depending on the type of cultivar [47,48]. The composition of citrus flavonoids varies considerably depending on the genetic origin of the fruit, the time of fruit harvest, and parts used [49,50].

#### 3.1.3. Naringin Content

**Figure 4:**
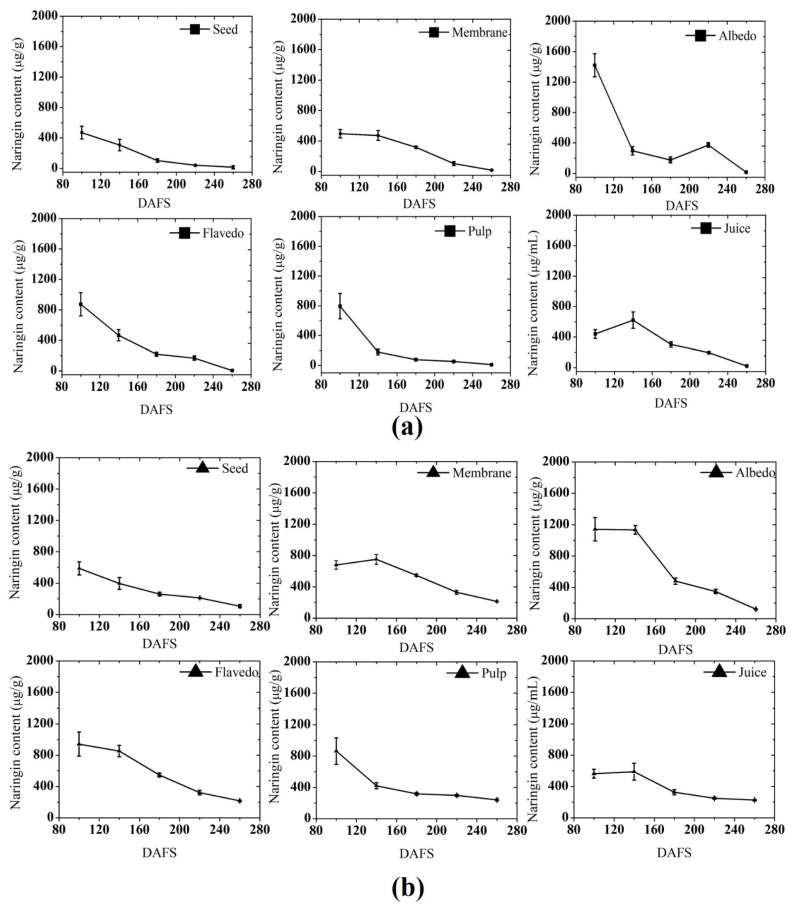
Naringin content estimated by HPLC in (**a**) 2018; and (**b**) 2019. DAFS; days after fruit set.

In grapefruit and pomelo, the main bitter compounds are limonin and naringin, which contribute to the unpleasant taste and reduce the acceptability among consumers [51]. Figure 4 presents the naringin content in various parts of the fruit at various maturity stages. The albedo contained the highest content of naringin while the seed had the lowest naringin content among different parts of pomelo fruit in the two seasons. The content found in the seed was highest at 100 DAFS in both seasons. However, the change pattern was quite similar in both seasons. At the beginning of the development stage, the content was highest, which decreased until the harvest.

An almost similar trend of naringin change was observed in the membrane, and the highest content was detected by 100–140 DAFS in both seasons. Naringin content presented a generalized decreasing trend until the harvest except for a little increase at 220–240 DAFS. However, a significant drop was observed until the harvest in both seasons. In 2018, the accumulation of naringin started to decrease from 100 DAFS up to the harvest phase, while in 2019, from 140 DAFS onwards, the accumulation consecutively decreases. 

Comparing the two seasons, 2019 had 7% more naringin content than 2018 in flavedo. In the pulp, the early stage of development accumulated the maximum amount of naringin in 2018 and 2019, and subsequently declined until the harvest stage.

The highest naringin content was registered on 100 DAFS (794 μg g^−1^ and 864 μg g^−1^) in both seasons during fruit development. When compared to the level at harvest stages, only 6.7% and 34.4% of naringin were left in the respective year. In both seasons, 2018 had the highest content of naringin in the juice at 140 DAFS with a level of 621 μg g^−1^, which was 5.3% higher than the consecutive season. The rate of change in the bitter compound naringin was quite similar in both seasons where the peel followed by the pulp had greater accumulation during the fruit growing period.

As reported by McIntosh and Mansell (1997) [51], the distribution of bitter compounds are tissue-specific, and naringin has the tendency to increase towards the albedo and flavedo portion of the fruit. The net effect of various processes, such as the transport of precursors from other plant tissues and the enzymatic activities of the in situ metabolism, influences the naringin distribution. Furthermore, the production of specific compounds, such as limonin glucoside, is related to the net production of limonoid aglycones during fruit maturation[52]. In addition, the level of limonin and nomilin in the fruit tissues was observed to increase in the early maturity and subsequently decreases and reaches a lower level [53]. Li et al. (2014) [54] noted a similar change in bitter compounds in Satsuma mandarin during the growth of fruit consecutive for three seasons. In the present study, the concentrations of naringin (in peel) were found to be higher than those reported by Li et al. (2014) [54] in both seasons.

Despite the general declining trend, fluctuations in the bitter compound were evidently large in some parts, including peel, membrane, and pulp, within two years. Hence, the data showed in the present study highlighted a similar consistency with the previous reports [53,54,55].

Castillo et al. (1992) [56] revealed a similar distribution for bitter compounds and suggested different relationships between the respective enzymatic activities in their biosynthetic pathway.

In 2019, the naringin level was low in all parts compared to the previous season, which could be attributed to the existence of a weak sink of bitter compounds, or strong naringinase activity, which catalyzes the conversion of naringin to naringenin.

#### 3.1.4. Tannin Content

**Figure 5:**
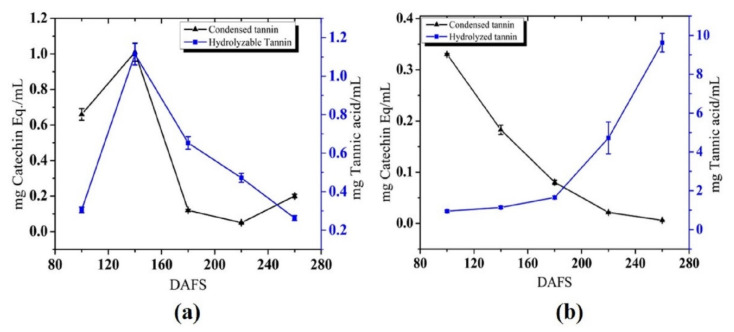
Tannin content in juice in (**a**) 2018; and (**b**) 2019. DAFS; days after fruit set.

**Figure 6:**
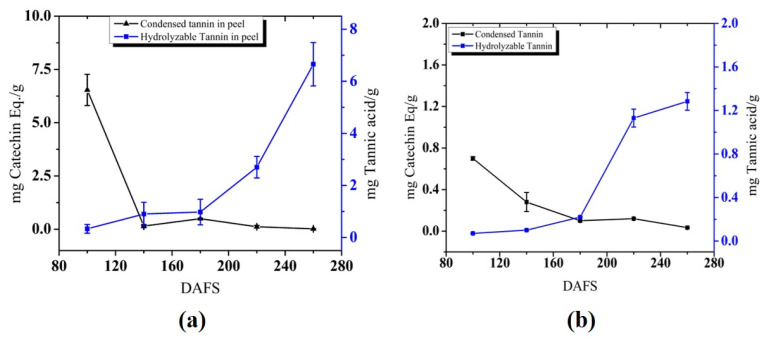
Tannin content in peel in (**a**) 2018; and (**b**) 2019. DAFS; days after fruit set.

Tannin is said to have astringent qualities; this is due to the fact that it binds to salivary proteins, resulting in a taste that humans perceive as astringency [57]. The tannin content of juice and peel was evaluated in terms of condensed and hydrolysable tannins and results are presented in Figure 5 and Figure 6 for both seasons. In the juice, both the condensed and hydrolysable tannins have shown a similar trend in 2018, but they differ in 2019. In 2018, the highest condensed tannin was seen at 100 DAFS (6.53 mg catechin) in the peel while 1.01 mg catechin mL^−1^ was observed in the juice at 140 DAFS. The lowest condensed tannin was recorded at 260 DAFS (0.02 and 0.2 mg catechin in peel and juice, respectively). On the other hand, the hydrolysable tannin in peel was highest at 260 DAFS (6.65 mg tannic acid) and lowest at 100 DAFS (0.33 mg tannic acid), while juice had the highest amount at 100 DAFS (1.11 mg tannic acid). Hydrolysable tannins gradually increased and then slowly decreased, as shown in Figure 5. Diba et al. [58] reported the decreased value of tannin with an increase of maturity of fig fruit. Astringency decreases with maturation, generally accompanied by a drop in tannin content or physicochemical changes in the molecules. Maturity causes tannins to polymerize due to the action of acetaldehyde, which is converted into sugars or consumed during respiration [59]. The tannins are present in the fruits in the form of non-polymerized tannin and these compounds tend to convert into a condensed form, ensuring more stability with proteins. This explanation portrays the decreased value of condensed tannin (astringency) in the fruit during growth and development [60].

However, in 2019, the condensed tannin in peel and juice was noticeably higher for first the few maturity stages and then decreased slowly showing the lowest at 260 DAFS (0.03 mg catechin g^−1^ and 0.006 mg catechin mL^−1^) (Figure 5 and Figure 6). The condensed tannin was lower than the previous season. The variation in the tannin content in pomelo peel and juice could be attributed to variety, environmental factors, and harvesting practices. More notably, the irrigation practices also affect the accumulation of tannin in the fruit [61]. However, the hydrolysable tannins showed a gradual increase, being lowest in immature fruit and highest in mature fruit. The hydrolysable tannin in peel was increasing gradually to 260 DAFS (1.28 mg tannic acid). The increase in hydrolysable tannin was 0.07 to 1.28 mg tannic acid g^−1^ throughout the development of fruit peel. The maximum tannin value in the juice was seen at 260 DAFS (9.62 mg tannic acid mL^−1^). In contrast, the hydrolysable tannin level in peel was shockingly low in comparison to the previous season, which has to be investigated further.

### 3.2. Ferric Reducing Antioxidant Power, DPPH Free Radical Scavenging Activity, and Antioxidant Capacity

There are many factors that influence the antioxidant potential of plant extracts, including the extraction method, the composition of the extract, and the maturity stages of the fruits. As we already know that there are several reactive oxygen species, such as hydroxyl radicals, superoxide radicals, hydrogen peroxides, lipid peroxides, and metal chelators, which, if present in the human body, may cause a number of complications. There is no single method available that may judge the potential of components to scavenge hydroxyl radicals, superoxide radicals, hydrogen peroxides, lipid peroxides, and metals simultaneously. Therefore, we chose three different techniques (i.e., free radicals scavenging, antioxidant capacity, and reducing power) to measure the capability of antioxidants found in pomelo, [3].

#### 3.2.1. Antioxidant Capacity by the Phosphomolybdenum Method

**Figure 7:**
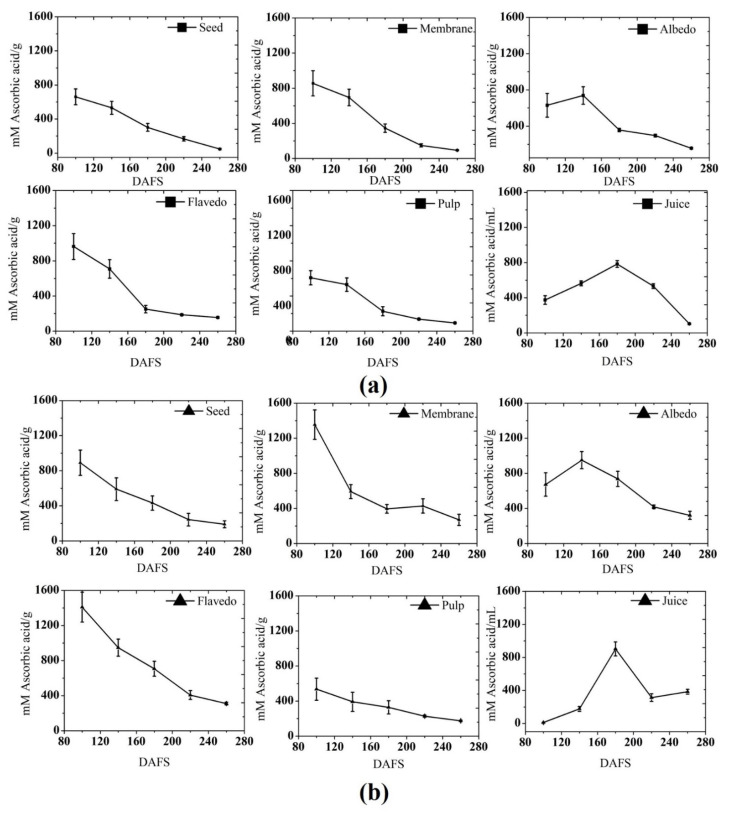
Antioxidant capacity by the phosphomolybdenum method in (**a**) 2018; and (**b**) 2019. DAFS: days after fruit set.

The antioxidant capacity in different parts of pomelo fruit at various maturity stages was calculated by the phosphomolybdenum method (Figure 7). The antioxidant capacity of citrus juice has been reported by several studies. It can be clearly seen from the result that seed, membrane, and pulp showed the same pattern of change in antioxidant activity. In the present study, the content changes as the tissue matures. The highest antioxidant capacity was noticed in the flavedo in 2019, which was 36.70% higher than the previous season and in other tissue. In the seed, a noticeable change in the antioxidant capacity was observed during the fruit development. Fruits harvested in the beginning stage had significantly higher antioxidant capacity than the later stages, with nearly three-fold higher antioxidant capacity compared to the harvest stage in both seasons. The antioxidant capacity decreases as the fruit attain maturity by September and starts ripening. A similar pattern of change in the antioxidant capacity was noticed in the membrane in 2018 and 2019, and interestingly, similar levels of antioxidant capacity were detected in both seasons. In the albedo, highest antioxidant capacity was observed in 2019 with a level of 950 mM AA g^−1^ at 140 DAFS, 22.31% higher than 2018, and then significantly decreased in the mature stage (August–September). Maximum antioxidant capacity was noticed in the beginning stage in the flavedo in 2018 and gradually decreased until harvest. Interestingly, the pattern of antioxidant capacity changes in the pulp was quite similar, and the highest antioxidant capacity was observed at 535 mM AA g^−1^ at 100 DAFS in 2018, which was the highest in both seasons. The antioxidant capacity encountered at the harvest stage was 32 and 30% in 2018 and 2019, respectively. A gradual but continuous increasing pattern was noticed in the juice until 180 DAFS, when the maximum antioxidant capacity was spotted at 180–200 DAFS in both seasons in the juice. However, thereafter, antioxidant capacity was decreased as the fruit attains ripening. The antioxidant capacity was increased up to 13% compared to the previous season. The observed changes in the antioxidant capacity of fruit could be related to the content of phenolic and flavonoids, which also decreased significantly during the growth and development stages of fruit. The results of the present study would confirm that there is a positive correlation between TPC and TFC and the antioxidant capacity, in agreement with previous studies [47]. The antioxidant capacity of the extract depends on non-enzymatic antioxidants, and a few enzymes, such as catalase, ascorbate peroxidase, and superoxidase dismutase activities, can scavenge free radicals [62]. Therefore, in pomelo parts, both systems (enzymatic and non-enzymatic antioxidants) might have contributed to the antioxidant capacity (AC) and variation in their level, fluctuating the AC of pomelo during maturation.

#### 3.2.2. Ferric Reducing Antioxidant Power (FRAP)

**Figure 8:**
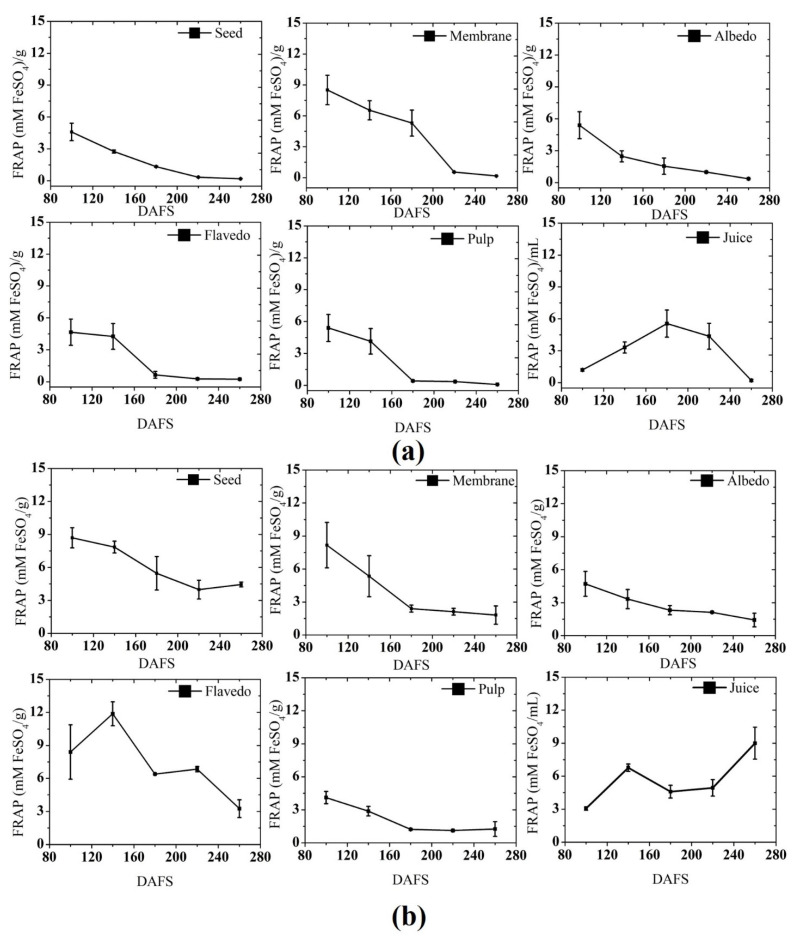
Ferric reducing antioxidant power in (**a**) 2018; and (**b**) 2019. DAFS: days after fruit set.

A FRAP assay was conducted for different parts of pomelo fruit during growth and developmental stages and the results are presented in Figure 8. The highest FRAP activity was observed in the fruit flavedo in 2019, with a level of 11.87 mM FeSO_4_ g^−1^, while the lowest activity was observed in the seed in 2018 (4.59 mM FeSO_4_ g^−1^). However, the declining pattern was quite similar in both seasons. A regular increase in FRAP activity was observed in the seed, where maximum activity was detected at 100 and 160 DAFS in 2018 and 2019, respectively, but there was a significant difference between the FRAP activity in 2018 and 2019. In the membrane, the maximum activity was noticed on 100–120, with a level of 8.16 mM FeSO_4_ g^−1^, followed by continuous degradation in the activity until the harvest stage. In the albedo, the pattern of change in FRAP activity was almost similar, where both seasons presented maximum activity at 100–120 DAFS. The change in FRAP activity in the fruit flavedo was quite different than the other tissue throughout the development, where in 2018 the highest value (11.87 mM FeSO_4_ g^−1^) was observed in the beginning stage, while the recorded activity in 2019 was 4.65 mM FeSO_4_ g^−1^. Thereafter, a systematic decrease in activity was seen until the harvest. In the pulp, both seasons showed a similar trend of change in the FRAP activity, where the highest activity was noticed at the beginning stage and the lowest at the harvest stage. However, a sharp decline phase appeared in 2018 at 160–200 DAFS, with a consecutive declining pattern in 2019. The fruit pulp harvested in 2019 had the highest FRAP activity, which was 62% higher than the previous season. The fruit harvested in 2018 had lower activity in the juice, but a systematic increased trend was observed until 180 DAFS, and afterward, the activity decreased until the harvest stage. In 2019, the increasing pattern was quite similar, except for the highest value, which was noticed at 260 DAFS. The juice obtained from the fruits harvested in 2019 (8.99 mM FeSO_4_ mL^−1^) had accumulated maximum FRAP activity at the harvest stage. The result obtained in the present study is supported by Zainudin et al. (2014) [6], who reported similar results: the activity was drastically decreased from 40.5% to 15.3%. The results reported by Menichini et al. (2009) [63] on pepper are in agreement with the present study. To the best of our knowledge, there are no studies on the antioxidant activity of pomelo fruit during the growth and developmental stage. The decreased activity during the ripening and overripening stages was attributed to a decrease in TPC and major individual phenolic compounds such as flavonol and hydroxycinnamic acid [64]. In the present study, the antioxidant activity was higher than that reported by Namiesnik et al. (2013) [65], as measured by FRAP.

#### 3.2.3. DPPH Free Radical Scavenging Activity

**Figure 9:**
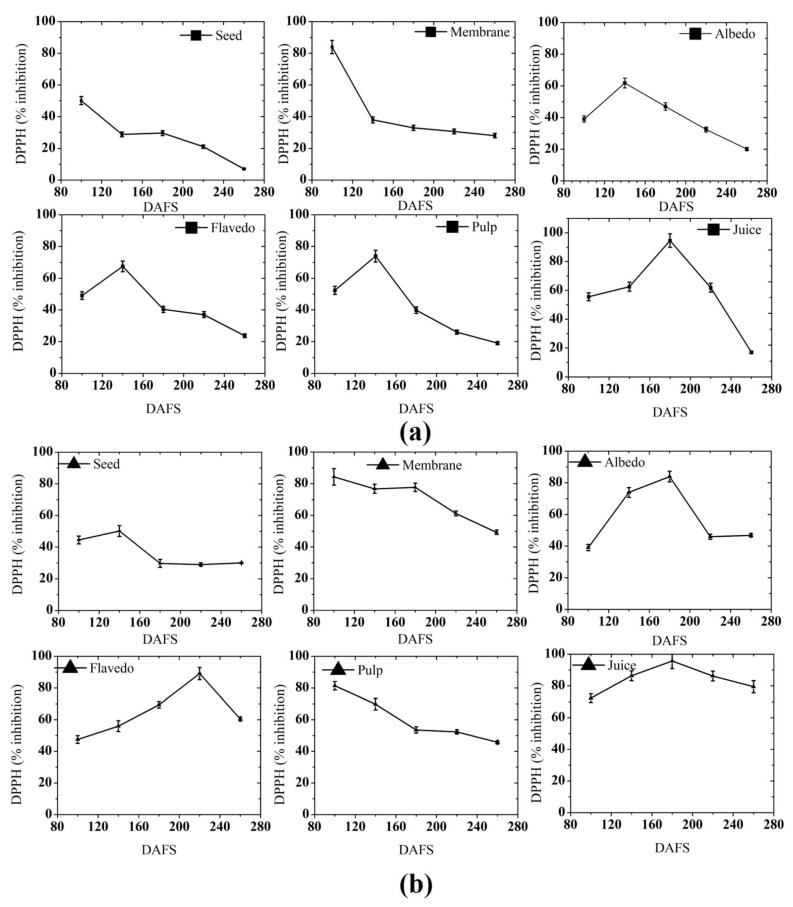
DPPH free radical scavenging activity in (**a**) 2018; and (**b**) 2019. DAFS: days after fruit set.

The radical scavenging activity of pomelo fruit at various maturity stages was calculated by DPPH and is presented in Figure 9. Juice presented the highest DPPH activity among all the parts of pomelo in 2018 and the order of the activity is juice > membrane > pulp > flavedo > albedo > seed. The seed of the pomelo fruit have shown a gradual increasing and then decreasing pattern in both seasons. The highest inhibition (50%) was observed at 100 DAFS and 140 DAFS for 2018 and 2019, respectively. For both seasons, the lowest inhibition was seen at 260 DAFS. In the membrane of pomelo fruit, the maximum inhibition was seen at 100 DAFS (84%). The inhibition activity of membrane started to decrease gradually. The albedo part of pomelo fruit presented a gradual but consistent increase in inhibition activity, which then decreased slowly in both seasons. The maximum inhibition by albedo was shown at 140 DAFS in 2018 (61%) and 180 DAFS in 2019 (83%). In flavedo, the trend was different in both the seasons. For the season of 2018, there was an increase in the inhibition activity up to 140 DAFS (89%), which then decreased sharply until 180 DAFS, showing a gradual decrease after that. The inhibition activity in flavedo in 2019 increased slowly, reaching the highest value at 140 DAFS (67%), after which it decreased. Pulp of pomelo at various maturity stages presented a consistent trend of increase and decrease. The maximum inhibition was registered at 140 DAFS and 100 DAFS (73 and 81, respectively), while the minimum inhibition was registered at the immature and overripe stages in 2018 and 2019, respectively. At the immature stage, the inhibition activity was decreased, while in the half mature stage, it was at peak level. A lower inhibition activity was observed at the mature and over-mature stages. Juice of pomelo fruit in both seasons presented a gradual but significant increase in inhibition activity until 180 DAFS, thereafter decreasing until reaching the over-mature stages. The maximum inhibition was observed at 180 DAFS (94–95%), while the minimum inhibition was observed at 260 DAFS. In general, the fluctuation in DPPH activity could be easily associated with the content of total phenol, ascorbic acid, and flavonoid, as supported by the observations of Ghasemi et al. (2009) [66] and Mansour (2019) [47]. We observed that the DPPH activity was proportionate with phenols and flavonoids (except in some tissues), which suggests a direct relationship between them. It has been reported that hydroxyl groups could contribute to the scavenging activity, but the plant phenols and flavonoids contained in the extract instead can reduce the scavenging activity due to their ability to donate hydrogen. In contrast, the plant flavonoid scavenging capacity and activity increased with an increase of the hydroxyl group and decreased in their glycosylation [67]. Muda et al. (2012) [68] observed that higher flavonoid content in the extract may contribute to the maximum free radical scavenging activity.

### 3.3. Variation in Volatile Composition of Pomelo Juice

Volatile compounds present in the pomelo juice of various maturity stages have been extracted using dichloromethane and have been identified by GC-MS. However, due to the unavailability of standards of volatile and aromatic compounds, the concentration of the volatiles and aromatic compounds of juice could not be authenticated, and therefore, they could not be presented here. The volatiles are categorized into hydrocarbons, esters, acids, alcohols, aldehydes, and other compounds. In the present study, a total of 111 volatiles was identified where 99 were present in 2018 and 101 compounds in 2019, respectively (Table 1). However, 91 compounds were common in both seasons. Citrus-based flavors represented by hydrocarbons (38 compounds) were found in the juice, which includes limonene, pinene, terpinene, copaene, elemene, humulene, etc. In 2018, 31 compounds were identified, whereas 34 hydrocarbons were identified in the consecutive season. During the growth and developmental stage of fruit, only three esters were identified in both seasons, which included neryl acetate, ethyl palmitate, and methyl esters, which are important for citrus aroma [13]. In contrast, 16 acid compounds were identified, and 14 compounds were common to both seasons. Important acids are nonanoic acid (responsible for rancid odor), butyric acid, linoleic acid, and cis-vaccenic acid (a precursor of rumenic acid). Vaccenic acid is useful in certain ailments, including cardiovascular disease, immune function, and inflammation. Aldehydes are a critical compound responsible for the characteristics citrus flavor [68].

In both seasons, 22 compounds were found, in which saturated aliphatic aldehydes (octanal and decanal), known for peely and citrus-like notes, were noted as potential candidates for citrus flavor [13]. Neral and geranial (monoterpenic aldehydes) were also observed. Aldehydes contribute to an intense green color, and the waxy odor was also found in the juice [13]. These compounds allow distinguishing pomelo from other citrus varieties [11]. During the growth of pomelo, 13 alcoholic volatile compounds were detected in the juice. Linalool (a monocyclic terpenoid) is responsible for the pleasant aroma of citrus. Neorl and geraniol (monoterpenoid alcohol) are important fragrant compounds that were also identified in the juice, having insecticidal and repellant properties. Volatile compounds, including nootkatone, quinine, indole, copaene, cadinene, etc., were categorized as other aromatic compounds. Nootkatone is a sesquiterpene ketone with a grapefruit-like aroma with a woody and bitter taste [13]. Apart from its aromatic character, nootkatone has also been reported to reduce the somatic fat ratio [69]. Thus, it can be assumed that pomelo could be a sustainable source for nootkatone production, which is the potential candidate for flavor, cosmetic, and fragrance industries [13]. Apart from nootkatone, there were 17 other volatile compounds identified that also play a significant role in the aromatic profile of pomelo fruit.

The concentration of volatiles changed significantly during the maturation process of pomelo fruit in both seasons [70]. Major hydrocarbons, including limonene, terpinene, pinene, and citral, represent approximately 80–86% of the total volatile compounds. These above-mentioned compounds have reached the maximum level at the end of the maturation process, while sesquiterpenes decreased gradually during the development process of fruit. The similar behavior of volatile compounds during growth has also been reported by Ledesma-Escobar et al. (2018) [71]. They reported on the variation of volatiles in Persian lime during the maturation process, and observed that 107 metabolites can be identified that experience significant changes during the growth phase, which caused clear, remarkable differences during the growth stages of lime.

### 3.4. Correlation of DAFS with Bioactive Compounds and Antioxidant Capacity

The correlation of DAFS with bioactive compounds and antioxidant capacity during different ripening stages showed a statistical significance level of 5% and 1%. These findings mirrored the trend reported for all the studied bioactive molecules (naringin content, FRAP, total phenolic content, total flavonoid content, antioxidant capacity, DPPH free radical scavenging activity), and most of them were found highest either in an immature or ripe stage in both the seasons (Supplementary Tables S1–S6). In general, the correlation of DAFS with bioactive compounds and antioxidant capacity were mostly negatively correlated in both seasons and significant at both levels (*p* < 0.05 and 0.01). This pattern suggests that all the bioactive compounds and antioxidant activities decreased when the fruit reached maturity.

In the case of seed, bioactive compounds were negatively correlated with DAFS in both seasons, while correlation among the bioactive compounds was positive at both the significance level. Few attributes, including FRAP with DPPH, antioxidant capacity and naringin, displayed a R^2^ ≥ 0.91. TPC had a stronger correlation with antioxidant, naringin, and TFC. In contrast, DAFS was negatively correlated with all the attributes, suggesting a decreasing pattern with maturity.

A similar pattern of change was noticed in the membrane in 2018, where the correlation of DPPH with other bioactive compounds was not significant except TFC with a R^2^ = 0.933. On the other hand, all the bioactive attributes in 2018 showed a significant correlation with each other, while fruits harvested in 2019 showed a weaker correlation. DAFS had a negative relation with all the attributes with a R^2^ ≥ −0.71.

An unusual relation was noticed in albedo, where in 2018, TPC and TFC had a strong correlation with DPPH, naringin with FRAP (R^2^ = 0.943), and TFC with DPPH (R^2^ = 0.993) and antioxidant activity (R^2^ = 0.884). TPC had a weaker relation with antioxidant capacity, FRAP, and naringin, indicating a decreasing pattern with an increase of TPC. In the case of 2019, poor correlation was seen in DPPH with bioactive compounds. FRAP established a strong correlation with naringin (R^2^ = 0.926), TFC (R^2^ = 0.991), and DAFS (R^2^ = −0.964). Similarly, naringin also had a strong relation with TFC, TPC, and DAFS.

Flavedo is rich in essential oils and certain bioactive compounds. Fruits grown in 2018 had a strong correlation of antioxidant capacity with FRAP (R^2^ = 0.982), naringin (R^2^ = 0.968), and TPC (R^2^ = 0.984), whereas FRAP was positively correlated with naringin (R^2^ = 0.909) and TPC (R^2^ = 0.957). Naringin was strongly correlated with TPC (R^2^ = 0.984). In 2019, developmental stages of pomelo fruit significantly affected the behavior of bioactive compounds, where DPPH was adversely affected as all the attributes were negatively correlated with maturity stages. Antioxidant capacity and naringin presented a strong correlation with TFC and TPC.

Pulp of fruit had only few significant correlations in 2018, such as antioxidant capacity with FRAP (R^2^ = 0.985), TFC (R^2^ = 0.905), and TPC (R^2^ = 0.920), while naringin was strongly linked with TFC (R^2^ = 0.883) and TPC with TFC (R^2^ = 0.992). In 2019, DPPH established a strong correlation with antioxidant capacity (R^2^ = 0.964), FRAP (R^2^ = 0.973), and naringin (R^2^ = 0.925). Antioxidant capacity with FRAP (R^2^ = 0.919) and naringin (R^2^ = 0.921) secured a strong correlation throughout the developmental stage.

In the case of juice, parameters such as antioxidant capacity, FRAP, TFC, and TPC were strongly correlated with DPPH, while the antioxidant capacity represented a correlation with FRAP, TFC, and TPC in 2018. On the contrary, only TPC was strongly linked with TFC (R^2^ = 0.992), while other attributes were not significant or had a weaker correlation in 2019.

## 4. Conclusions

Pomelo contains appreciable nutrients and bioactive compounds and the change in volatile and bioactive components with maturity stages is a complex phenomenon. Thus, harvesting pomelo fruit at optimum levels will result in fruit with optimal health beneficial compounds. From the presented results, we can assume that among all the parts of pomelo, the highest total phenol, total flavonoid, and naringin content can be found in the membrane part in both seasons. The antioxidant capacity and FRAP value were highest in the flavedo part of the pomelo. The juice possessed the maximum amount of DPPH and tannin content. As less literature is available on the phytochemical and flavor profiling of pomelo, these results will be useful for pomelo processing and product development. In addition, variation in volatile compounds during maturity will also help to harvest the fruits at the maximum concentration of volatile compounds. The best time for pomelo harvesting was observed to be 180–220 DAFS based on the results of growth characteristics, physical parameters, chemical composition, sensorial attributes, and bioactive compounds. This suggested range showed the maximum health benefitting attributes, such as the total phenolic content, flavonoid content, DPPH free radical scavenging activity, etc., along with appreciable chemical compounds, such as titrable acidity, ascorbic acid, and sugar content. The peel color value (L*, a*, and b*) also reached the maximum at this stage.

Thanks to its impressive and diverse properties, fruit can be used for the production of high-quality food products and health-beneficial supplements, where selecting the right fruit harvesting stage is crucial.

Data provided in this study will be crucial for fruit growers and processors to select the optimal harvesting period for pomelo. However, sensory evaluation and nutritional quality of fruits at various maturity stages could be useful to generate more reliable information for future developments.

## Figures and Tables

**Table 1:** Overall compounds identified in pomelo juice extracts harvested in 2018 and 2019.

S. No.	Overall Compounds	Identified in 2018	Identified in 2019
**Hydrocarbons**			
1.	α-pinene	+	+
2.	β-pinene	+	+
3.	Sabinene	+	+
4.	β-Myrcene	+	+
5.	α-terpinene	+	+
6.	Camphene	+	+
7.	Cyclohexane	+	+
8.	m-cymene	+	+
9.	Benzene	+	+
10.	Dodecane	+	+
11.	9-tricosene	+	−
12.	Caryophyllene	−	+
13.	Alloaromadendrene	−	+
14.	α-Copaene	+	+
15.	β-Copaene	+	+
16.	α,β-Cubebene	+	+
17.	Limonene	+	+
18.	17-Acetyloxy	+	+
19.	β-Phellandrene	+	+
20.	trans-β-Ocimene	−	+
21.	cis-β-Ocimene	−	+
22.	q-Cymene	+	+
23.	Terpinolene	+	+
24.	4,8-Dimethyl-1,3,7-nonatriene	+	−
25.	d-Elemene	+	+
26.	γ-Elemene	+	+
27.	β-Caryophyllene	−	+
28.	c-Elemene	+	+
29.	α-Humulene	+	+
30.	β-Humulene	+	+
31.	Germacrene D	+	−
32.	α-Selinene	+	+
33.	1,1-Biphenyl	+	+
34.	z,z,z-4,6,9-Nonadecatriene	+	−
35.	Coumarin	+	+
36.	Longifolene	−	+
37.	Cyclohexene	+	+
38.	4-Carene	−	+
**Esters**			
39.	Neryl acetate	+	+
40.	Ethyl palmitate	+	+
41.	Methyl esters	+	+
**Acids**			
42.	Acetic acid	+	+
43.	Malonic acid	+	+
44.	2-Pentenoic acid	+	+
45.	Propionic acid	+	+
46.	Hexadecanoic acid	+	+
47.	Nonanoic acid	+	+
48.	Butyric acid	+	+
49.	Benzoic acid	+	+
50.	Oleic acid	+	+
51.	Linoleic acid	+	+
52.	α-Linoleic acid	+	+
53.	Oxalic acid	+	+
54.	1,2,3,4-Tetrahydroisoquinolin-6-ol-1-carboxylic acid	+	−
55.	Mercaptobenzoic acid	+	−
56.	4-Oxatricyclo[6.3.0.0]-undecan-5-one-1-carboxylic acid	+	+
57.	Cis-vaccenic acid	−	+
**Alcohols**			
58.	Phenol	+	+
59.	Ethanol	+	+
60.	Ergoline 8-Methanol	−	+
61.	1-dodecanol	+	+
62.	Linalool	+	+
63.	Nerol	+	+
64.	Geraniol	+	+
65.	Carveol	+	+
66.	1-Heptatriacotanol	+	+
67.	Eugenol	+	+
68.	1-Nonadecanol	+	−
69.	1-Undecanol	+	−
70.	Corynan-17-ol	+	+
**Aldehydes**			
71.	Octanal	+	+
72.	7-Heptadecene	+	+
73.	Decanal	+	+
74.	Benzaldehyde	+	+
75.	Citronellal	+	+
76.	Neral	−	+
77.	Geranial	+	+
78.	trans,cis-2,4-Decadienal	+	+
79.	Dodecanal	+	+
80.	Perilla aldehyde	+	+
81.	trans,trans-2,4-Decadienal	+	−
82.	trans-2-Dodecenal	+	−
83.	cis,trans-2,6-nonadienal	+	+
84.	Octadecanal	+	+
85.	9-Octadecenoic acid	+	+
86.	2-Nonadecanone	+	+
87.	D-carvone	+	+
88.	4H-1-Benzopyran-4-one	+	+
89.	2,4-Cycloheptadien	−	+
90.	2,6-dihydroxyacetophenone	+	+
91.	9,10-Anthracene-dione	+	+
92.	3H-cycloocta[c]pyran-3-one	+	+
93.	Flavone	+	+
**Others**			
94.	Limonene oxide	+	+
95.	3,5-Androstadien-17-one-oxime	+	+
96.	δ-Cadinene	+	+
97.	Bis(trimethylsiyl) ether	+	+
98.	Trienbolone	+	+
99.	(-)-Isolongifolol	+	+
100.	Silane	+	+
101.	Di-Silane	+	+
102.	Copaene	+	+
103.	Bicyclo	+	+
104.	1H-indole	+	+
105.	Chinchonan	+	+
106.	Giseofulvin	+	+
107.	Quinine	+	+
108.	Indole	+	+
109.	Nootkatone	+	+
110.	Osthole	+	+
111.	3-Piperdinamine	+	+

Note: + (present); − (absent).

## Data Availability

The data presented in this study are available on request from the corresponding author.

## Supplementary Materials

The following are available online at https://www.mdpi.com/article/10.3390/plants10091941/s1, Table S1: Correlation of DAFS with bioactive compounds and antioxidant activities in seed; Table S2: Correlation of DAFS with bioactive compounds and antioxidant activities in membrane; Table S3: Correlation of DAFS with bioactive compounds and antioxidant activities in albedo; Table S4: Correlation of DAFS with bioactive compounds and antioxidant activities in flavedo; Table S5: Correlation of DAFS with bioactive compounds and antioxidant activities in pulp; Table S6: Correlation of DAFS with bioactive compounds and antioxidant activities in juice.
