# Supplementary material for: Variation in Phytochemical, Antioxidant and Volatile Composition of Pomelo Fruit (Citrus grandis (L.) Osbeck) during Seasonal Growth and Development
Source: Plants (Basel). 2021 Sep 17;10(9):1941. doi: 10.3390/plants10091941 (PMC8467822; doi:10.3390/plants10091941)
Supplement: Supplementary file 1 [file plants-10-01941-s001.zip › plants-1357440-Supplementary.pdf]

Table S1. Correlation of DAFS with bioactive compounds and antioxidant activities in seed

2018	DPPH	Antioxidant capacity	FRAP	Naringin	TFC	TPC
Antioxidant capacity	0.917*					
FRAP	0.916*	0.972**				
Naringin	0.888*	0.974**	0.994**			
TFC	0.872	0.934*	0.978**	0.988**		
TPC	0.943*	0.938*	0.987**	0.969**	0.965**	
DAFS	-0.950*	-0.993**	-0.960**	-0.953*	-0.910*	-0.938*

Note: significance level: * p<0.05; ** p<0.01

2019	DPPH	Antioxidant capacity	FRAP	Naringin	TFC	TPC
Antioxidant capacity	0.779					
FRAP	.913*	0.957*				
Naringin	0.786	0.987**	0.933*			
TFC	0.500	0.864	0.738	0.870		
TPC	0.549	0.918*	0.795	0.918*	0.991**	
DAFS	-0.799	-.973**	-.936*	-.976**	-0.757	-0.832

Table S2. Correlation of DAFS with bioactive compounds and antioxidant activities in membrane

2018	DPPH	Antioxidant capacity	FRAP	Naringin	TFC	TPC
Antioxidant capacity	0.813					
FRAP	0.750	0.954*				
Naringin	0.678	0.961**	0.981**			
TFC	0.933*	0.947*	0.931*	0.894*		
TPC	0.871	0.984**	0.947*	0.944*	0.982**	
DAFS	-0.807	-0.975**	-0.968**	-0.974**	-0.962**	-0.990**

2019	DPPH	Antioxidant capacity	FRAP	Naringin	TFC	TPC
Antioxidant capacity	0.708					
FRAP	0.755	0.950*				
Naringin	0.925*	0.627	0.783			
TFC	0.927*	0.874	0.937*	0.923*		
TPC	0.783	0.459	0.467	0.709	0.708	
DAFS	-0.948*	-0.852	-0.919*	-0.938*	-0.998**	-0.710

Table S3. Correlation of DAFS with bioactive compounds and antioxidant activities in albedo

2018	DPPH	Antioxidant capacity	FRAP	Naringin	TFC	TPC
Antioxidant capacity	0.827					
FRAP	0.358	0.755				
Naringin	0.089	0.552	0.943*			
TFC	0.993**	0.884*	0.427	0.160		
TPC	0.965**	0.723	0.280	-0.022	0.942*	
DAFS	-0.681	-0.910*	-0.926*	-0.778	-0.728	-0.608

2019	DPPH	Antioxidant capacity	FRAP	Naringin	TFC	TPC
Antioxidant capacity	0.654					
FRAP	-0.167	0.595				
Naringin	0.095	0.814	0.926*			
TFC	-0.174	0.569	0.991**	0.901*		
TPC	0.420	0.909*	0.824	0.891*	0.812	
DAFS	-0.100	-0.773	-0.964**	-0.956*	-0.949*	-0.944*

Table S4. Correlation of DAFS with bioactive compounds and antioxidant activities in flavedo

2018	DPPH	Antioxidant capacity	FRAP	Naringin	TFC	TPC
Antioxidant capacity	0.733					
FRAP	0.822	.982**				
Naringin	0.645	.968**	.909*			
TFC	0.863	0.794	0.813	0.779		
TPC	0.759	.984**	.957*	.984**	0.813	
DAFS	-0.793	-.932*	-.905*	-.952*	-.930*	-.960**

2019	DPPH	Antioxidant capacity	FRAP	Naringin	TFC	TPC
Antioxidant capacity	-0.702					
FRAP	-0.287	0.646				
Naringin	-0.670	.961**	0.816			
TFC	-0.705	.990**	0.704	.968**		
TPC	-0.622	.949*	0.848	.989**	.973**	
DAFS	0.588	-.976**	-0.771	-.984**	-.971**	-.977**

Table S5. Correlation of DAFS with bioactive compounds and antioxidant activities in pulp

2018	DPPH	Antioxidant capacity	FRAP	Naringin	TFC	TPC
Antioxidant capacity	0.877					
FRAP	0.810	0.985**				
Naringin	0.437	0.812	0.850			
TFC	0.702	0.905*	0.862	0.883*		
TPC	0.737	0.920*	0.876	0.870	0.992**	
DAFS	-0.828	-0.957*	-0.912*	-0.823	-0.970**	-0.988**

2019	DPPH	Antioxidant capacity	FRAP	Naringin	TFC	TPC
Antioxidant capacity	0.964**					
FRAP	0.973**	0.919*				
Naringin	0.925*	0.921*	0.934*			
TFC	0.179	0.334	0.029	-0.049		
TPC	0.194	0.303	0.067	-0.092	0.972**	
DAFS	-0.960**	-0.986**	-0.888*	-0.859	-0.429	-0.411

Table S6. Correlation of DAFS with bioactive compounds and antioxidant activities in juice

2018	DPPH	Antioxidant capacity	FRAP	Naringin	TFC	TPC
Antioxidant capacity	0.982**					
FRAP	0.897*	0.948*				
Naringin	0.482	0.490	0.246			
TFC	0.905*	0.930*	0.791	0.759		
TPC	0.916*	0.938*	0.865	0.446	0.906*	
DAFS	-0.445	-0.360	-0.067	-0.872	-0.582	-0.281

2019	DPPH	Antioxidant capacity	FRAP	Naringin	TFC	TPC
Antioxidant capacity	0.833					
FRAP	0.050	0.077				
Naringin	-0.309	-0.553	-0.386			
TFC	0.633	0.220	0.092	0.446		
TPC	0.560	0.145	0.006	0.552	0.992**	
DAFS	0.257	0.415	0.696	-0.925*	-0.295	-0.412
